# Supplementary material for: A PALB2-interacting domain in RNF168 couples homologous recombination to DNA break-induced chromatin ubiquitylation
Source: eLife. 2017 Feb 27;6:e20922. doi: 10.7554/eLife.20922 (PMC5328590; doi:10.7554/eLife.20922)
Supplement: Supplementary file 1. — (A) List of siRNAs and (B) List of antibodies. DOI: http://dx.doi.org/10.7554/eLife.20922.021 [file elife-20922-supp1.docx]

**Supplementary File 1**

**(A) List of siRNAs**

| Target | Sequence |
| --- | --- |
| BRCA1 | AGAUAGUUCUACCAGUAAAUU |
| BRCA2 | GAAGAAUGCAGGUUUAAUAUU |
| Luciferase (Luc) | CGUACGCGGAAUACUUCGAUU |
| PALB2  (smartpool) | GUUUCAGAGUUAAAGAAUC  CCACUAUUCAUAAGUUAGG  CCAACUUGCUCAUUUGAAA  UUGUUAUUUGGAAUUUAAA |
| RAP80  (smartpool) | GTAAATCCCTGGTCCCATTUU  AAATGAATCTCCCGTCAAGUU  AGAGCAGGCTAGTGAGAAAUU  AGAGGCAGCTCCTTAATAAUU |
| RNF168-1 | GACACUUUCUCCACAGAUAUU |
| RNF168-3 | gaagagtcgtgcctactgaUU |
| RNF8-1 | GAGGGCCAAUGGACAAUUAUU |
| RNF8-2 | UGCGGAGUAUGAAUAUGAAUU |

**(B) List of Antibodies**

| Antibody | Host | Company (reference) | IF | WB |
| --- | --- | --- | --- | --- |
| 53BP1 | Rabbit | Novus (NB100-304) | 1:1000 |  |
| BRCA1 | Mouse | SantaCruz (sc-6954) | 1:100 |  |
| BRCA2 | Rabbit | Oncogene |  | 1:500 |
| Cyclin A | Rabbit | SantaCruz (sc-596) | 1:100 |  |
| Cyclin B1 | Mouse | SantaCruz (sc-245) | 1:100 |  |
| FK2 | Mouse | Enzo (BML-PW8810-0500) | 1:100 |  |
| FLAG | Mouse | Sigma (F1804) | 1:100 |  |
| FLAG | Mouse | Sigma (F3165) |  | 1:5000 |
| GFP | Mouse | Roche (11814460001) |  | 1:2000 |
| GST | Rabbit | Cell Signaling (2625) |  | 1:1000 |
| His | Mouse | Clontech (631212) |  | 1:2500 |
| mCherry | Mouse | Abcam (ab125096) |  | 1:500 |
| MDC1 | Rabbit | Abcam (ab11171-50) | 1:1000 |  |
| PALB2 (IF) | Rabbit | Gift of Dr. Bing Xia | 1:200 |  |
| PALB2 (WB) | Rabbit | Bethyl (A301-246A) |  | 1:1000 |
| RAD51 | Rabbit | SantaCruz (sc-8349) | 1:100 |  |
| RAP80 | Rabbit | Bethyl (A300-764) | 1:500 |  |
| RNF168 | Rabbit | Millipore (ABE367) | 1:200 | 1:500 |
| RNF8 | Mouse | SantaCruz (sc-271462) | 1:50 | 1:100 |
| RPA2 | Mouse | Abcam (ab2175) | 1:1000 | 1:1000 |
| Tubulin | Mouse | Sigma (T6199) |  | 1:5000 |
| Ubiquitin | Rabbit | VIVA Bioscience (K12082) |  | 1:1000 |
| γH2AX | Mouse | Millipore (clone JBW301) | 1:2000 |  |
